# Surgeon-Powered Robotics in Thoracic Surgery; An Era of Surgical Innovation and Its Benefits for the Patient and Beyond

**DOI:** 10.3389/fsurg.2020.589565

**Published:** 2020-11-26

**Authors:** Jason Trevis, Nicholas Chilvers, Kathrin Freystaetter, Joel Dunning

**Affiliations:** Department of Cardiothoracic Surgery, James Cook University Hospital, Middlesbrough, United Kingdom

**Keywords:** VATS, thoracic, robotic, wristed instruments, surgeon-powered robotics

## Abstract

Following its introduction in 1992, the growth of minimally invasive thoracic surgery was initially hampered by the lack of specialized instruments, impeded visualization and stapling. However, in subsequent years these challenges were somewhat overcome and video-assisted thoracoscopic surgery (VATS) became the preferred modality of many centers. More recently, robotic surgery has come to the fore. Whilst it offers outstanding precision via robotic wristed instruments, robotic surgery is expensive and has safety implications as the surgeon is away from the patient's side. Wristed VATS instruments offer a new, exciting alternative. By placing the robotic-like wristed instruments in the hands of the surgeon, a concept we call surgeon-powered robotics, the benefits of robotic surgery can be achieved by the patient's side. We describe our experience of the ArtiSential® wristed instruments and discuss the benefits and challenges of this technology. By combining wristed instruments with the latest surgeon-controlled 3D camera technology, surgeon-powered robotics is an affordable reality.

## Introduction

Following its introduction in 1992, the growth of minimally invasive thoracic surgery was initially hampered by the lack of specialized instruments, impeded visualization and stapling (1). However, in subsequent years these challenges were somewhat overcome and video-assisted thoracoscopic surgery (VATS) became the preferred modality of many centers. Such minimally invasive techniques have well-documented benefits for patients when compared to open procedures. This was highlighted most recently in the preliminary results of the VATS vs. conventional open lobectomy for lung cancer (VIOLET) trial, presented at the International Association for the Study of Lung Cancer 2019 World Conference on Lung Cancer (2). VATS afforded lower complication rates and reduced hospital stay, without compromise to oncological outcomes.

More recently, robotic surgery has come to the fore. Offering outstanding precision via robotic wristed instruments and paired with the latest in 3D camera technology, increasing numbers of centers are adopting this approach. However, this system comes at a price; a very high one. Not only do these systems cost an average of 2 million dollars, they also have safety implications, as the surgeon is un-scrubbed away from the patient's side and relies on assistants to perform key parts of the procedure.

Wristed VATS instruments offer a new, exciting alternative. By placing the robotic-like wristed instruments in the hands of the surgeon, a concept we call surgeon-powered robotics, the benefits of robotic surgery can be achieved by the patient's side. We discuss our experience of this state-of-the-art technology, particularly the ArtiSential® wristed instruments (LivsMed Inc., Gyeonggi-do, Republic of Korea), and discuss the wider reported advantages and challenges.

## A New ERA

The specialty of thoracic surgery is set to be transformed by lung cancer screening, which will lead to a dramatic shift in the type of patients and pathology that are seen. Enhanced recovery and minimally invasive surgery will also continue to evolve. This creates scope for the development of novel and hybrid technologies to best deal with such early cancers, most likely utilizing navigational bronchoscopy and targeted therapies, or complex resections for resistant tumors. We must therefore remain flexible as surgeons and embrace new technologies and approaches.

Despite the vast increase in the proportion of thoracic procedures carried out via a VATS approach, innovation in instrument technology remains relatively static despite continued unaddressed pitfalls. It could be argued that this is due to the more recent increase in the number of robotic cases performed, which require large financial investment despite their low cost-effectiveness.

To instigate progression within the speciality, the limitations of VATS must be recognized. At the heart of these limitations lie the key concepts of degrees of freedom, haptic feedback and ergonomics. There remains a limited degree of freedom in thoracoscopic surgery. Whereby six degrees represents free movement like that experienced in open surgery, standard endoscopic instruments allow for only four (rotation, in/out, left/right, up/down movements). Consequently, surgeons encounter situations where the angles of their instruments are suboptimal, with little flexibility or scope to address this issue. This is compounded by poor transfer of force due to a lack of ergonomically designed handles and poor haptic feedback (3). The challenges are further exemplified in the development of uniportal thoracic surgery. The closer proximity of the instruments and camera lead to reduced maneuverability and loss of triangulation. The surgeon must also frequently adopt uncomfortable body positions for prolonged lengths of time (4). Although the technical aspects of performing VATS have been refined somewhat, issues surrounding their ergonomics and surgeon fatigue must also be considered.

Whilst many of these issues were addressed with the da Vinci® Surgical System (Intuitive, Sunnyvale, California, USA), its introduction brought about a new set of difficulties, namely low cost-effectiveness and low clinical usage due to a small number of units at any given center. Consequently, there has been a desire for the development of instruments that harbor the benefits of robotic systems, with the retained cost-effectiveness and flexibility of VATS. Wristed instruments aim to deliver this; the ArtiSential® instruments boast a 360-degree angle of motion and six degrees of freedom, comparable to the da Vinci® Surgical System.

## Artisential® Wristed Instruments

**Figure 1:**
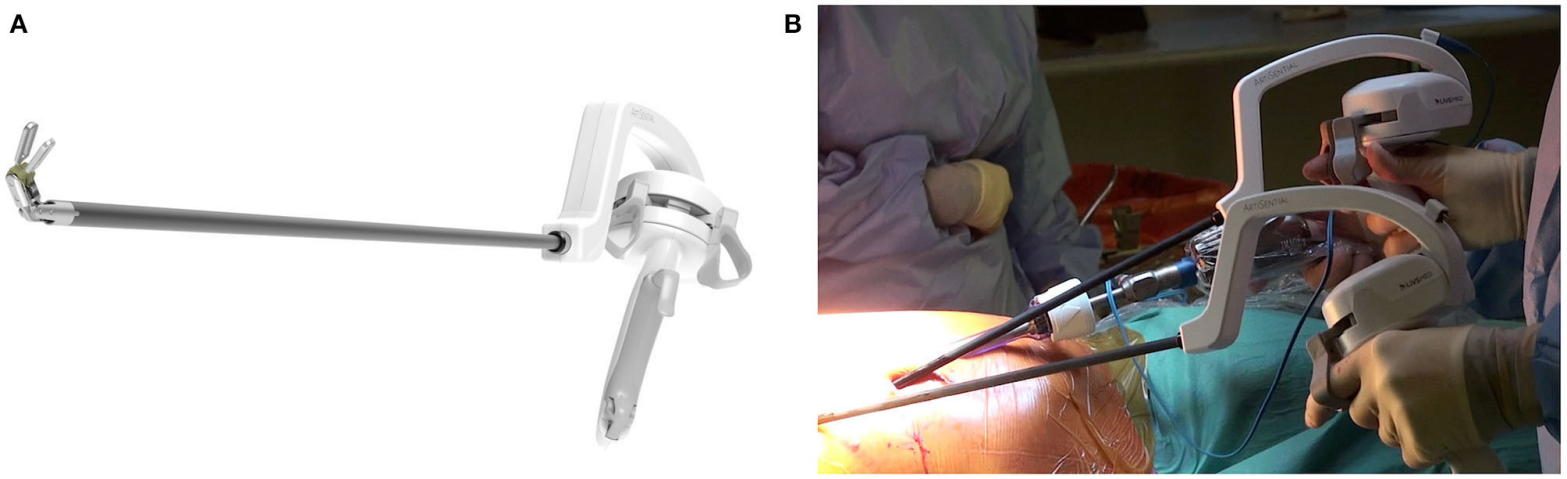
**(A)** ArtiSential® bipolar fenestrated forceps. **(B)** Intraoperative use of ArtiSential® instruments.

The Artisential® wristed instruments (Figure 1) have been developed by the South Korean company LIVSMED (*www.livsmed.com*). The range includes fenestrated forceps, bipolar fenestrated forceps, bipolar Maryland dissector, bipolar precise dissector, needle holder, precise needle holder, clip applier, monopolar spatula and monopolar hook. Although initially designed for abdominal surgery, LIVSMED has now engineered shorter instruments appropriate for thoracic surgery. Our center was the first to use these instruments for this purpose in January 2020.

The fully articulating wristed motion provides superior flexibility compared to standard VATS instruments. With movement of the surgeon's wrist left/right, up/down and rotating left/right, the tip of the instrument moves in the corresponding direction, as in robotics. Similarly, grasping tissues is achieved by pinching and releasing the fingers. However, gross movements of the surgeon's arm result in counterintuitive movements in the opposite direction due to the “fulcrum effect,” as experienced with conventional VATS instruments. A locking mechanism facilitates port entry and tissue handling. The instruments can be placed through 8 mm robotic ports or directly through the chest wall.

## Our Experience

To date, our center has performed six cases with the ArtiSential® wristed instruments across a diverse range of procedures. The supplementary material contains intra-operative video footage to highlight the benefits described.

### Right Lower Lobectomy (Supplementary Video 1)

Our usual set up (subxiphoid port, anterior camera port and 2 anterior utility ports) takes the same time as standard VATS cases and is quicker than robotics. The Maryland dissector can be easily manipulated to the ideal angle with simple wrist movements and the bipolar energy device facilitates coagulation of small vessels with less displacement of energy compared to monopolar devices. The ease of manipulation permits precise control during dissection, establishing the ideal tissue planes around the hilum. Supplementary Video 1 also demonstrates how easily instruments can be changed, for example to a sucker or standard diathermy, as the surgeon is at the bedside. Similarly, you can see the surgeon themselves, rather than an assistant, introducing the stapler around the inferior pulmonary vein. The specimen was removed through the subxiphoid port.

### Left Upper Lobectomy (Supplementary Video 2)

During this left upper lobectomy, the ability to angle the instruments to 90 degrees in all directions enabled accurate dissection of the pleura and vessels with the Maryland dissector and subsequently the fenestrated forceps. This feature is further appreciated when dissecting out the lymph nodes, with clear superiority to standard VATS instruments. The tactile feedback received from these instruments makes it possible for even the pointed Marylands to be used safely to dissect around the segmental pulmonary artery branch prior to stapling. We have also performed a right upper microlobectomy in a similar fashion.

### Thymectomy (Supplementary Video 3) and Thymectomy With Left SVC (Supplementary Video 4)

Successful thymectomy via a minimally invasive approach relies on achieving a complete resection without harm to vital nearby structures. Again, the operator can achieve perfect angles with the Maryland dissector. Supplementary Video 3 demonstrates how subtle sweeping movements of the wristed instruments facilitate dissection of the fat between the innominate vein and the superior vena cava (SVC).

A left sided approach was necessary for a more complex thymectomy case with a left sided SVC (Supplementary Video 4). The orientation achieved with the wristed instruments suits the tissue planes perfectly.

### Diaphragm Plication (Supplementary Video 5)

This procedure showcases the dexterity of the fully articulated instruments during suturing. The needle holder can be used to grasp the needle exactly in the desired place, whilst the second wristed instrument can safely grasp or retract the tissue. In this case 10–12 horizontal mattress sutures are placed in an anterolateral to posteromedial position. Our normal practice is to tie outside the chest and use the Chitwood Knot Pusher (Scalan International, *www.scanlaninternational.com*). However, we demonstrate here that knot tying a Weston knot, with subsequent wristed instrument-tied throws, is incredibly straightforward.

## Discussion

Table 1 summarizes the benefits and current challenges of wristed-instrument technology.

**Table 1:** Benefits and current challenges of wristed-instrument technology.

**Benefits**	**Challenges**
Lead surgeon at the bedside	Combination of VATS and robotic techniques
Surgeons performs the key steps e.g., stapling	Resistance of the chest wall in the port due to VATS style pivot point
Increased tactile feedback	Camera technology development/advancement
Greater precision	Operator experiences the fulcrum effect
More cost effective	
Less training time required	
Quicker procedures with increased flexibility	

### Advantages

When considering the use of wristed instruments, and drawing comparison to that of robotic thoracic surgery, the benefits can be broadly categorized into those impacting safety and cost. Many of the safety benefits of using these wristed instruments stem from having the lead surgeon at the bedside, rather than at a distance at a console. This enables visualization of the patient in their entirety, rather than from a digital isolated internal viewpoint. This allows for early management of any complications that may arise during the procedure, without the delay in waiting for the surgeon to “re-scrub.” This concept also acts to reduce potential barriers to communication between the healthcare team and the lead surgeon. The importance of such human factors has been particularly prudent during the COVID-19 pandemic, with personal protective equipment impacting on communication. Furthermore, from a technical standpoint, being positioned at the bedside ensures that the surgeon performs the key steps in the procedure, for example stapling, in comparison to most current robotic systems (with exception of the da Vinci® Xi Surgical System) that do not support stapling instruments. This notion is further improved by the tactile feedback gained through the use of wristed instruments, as opposed to robotics. In all, this contributes to increased precision when compared to VATS, due to the dexterity of a robotic instrument, with added tactile feedback. There are also ongoing attempts to develop technology for the integration of enhanced tactile sense (5). However, due to the wired articulating joint mechanism of the ArtiSential® wristed instruments, resistance from the tissue or tension during knot tying (6), can be sensed between the jaws of the instrument (5). This in contrast to the lack of haptic feedback exerted by the da Vinci® system.

As a center, elements which have been highlighted as key benefits include the added dexterity imparted by the wristed mechanism, which is above and beyond that of a rigid VATS instrument. This was particularly evident in the cases described. In addition, wristed instruments retain the flexibility and speed of VATS, especially when switching between instruments or ports. This increases the speed of the procedure when compared to undertaking the same process with a robot. Such flexibility is extended by the option to utilize both wristed and traditional instruments if desired. It may be that the surgeon wishes to exploit the increased dexterity of the ArtiSential® during a difficult case, when transitioning to the da Vinci® is simply unfeasible (5).

Aside from the technical benefits imparted by the use of such wristed instruments, they also replicate the advantages typically brought about by robotic instrumentation when compared to VATS techniques. The monetary investment required to run a robotics programme will be negated by the development of these new techniques, a vital consideration for those systems in which development of services is typically limited by scarce financial resources. The cost effectiveness of such wristed instruments is multifaceted when considering the wider financial cost of establishing a robotic programme. A key concept is the reduced training time required to initiate and run a wristed instrument programme (10–20 vs. 30 h for the robot), therefore increasing the number of staff competent in performing such cases, potentially improving the patient flow. Furthermore, even for those centers with an established robotic programme, it can be argued that the implementation of bedside wristed instrumentation brings about greater case throughput due to the lack of shared robotic sessions between specialties. The faster procedure time typically seen in VATS cases, when compared to robotics, further increases patient flow and reduces waiting times. With further technological advancement, additional cost saving methods may be imparted when using wristed instruments; namely, a robotic camera holder rendering an assist redundant (7). This is an element which may truly transform thoracic surgery, hybridizing the benefits of VATS and robotic surgery through the use of a three-dimensional surgeon-controlled camera, wristed instruments and tactile feedback with safety of the surgeon at the patient's bedside.

### Challenges

Despite the numerous aforementioned benefits, some challenges remain in relation to instigating practice with wristed instruments. Whilst the trocar pivot point and resistance of the chest wall restrict the degree of movement, which can result in localized trauma at the incision site or compression of the intercostal nerve, this is somewhat offset by the increased dexterity provided by the wrist mechanism. This results in a reduction in the need for extremes of movement that may be required using VATS instruments. Furthermore, the intuitiveness of operating with a robotic system is removed and the surgeon must adapt their technique, accounting for the fulcrum effect that VATS surgery imposes. This is arguably a minor challenge in the era of split practice between VATS and robotic surgery, whereby competency in both exists. This, however, raises an issue for surgeons who have not embraced robotic practice, in that the techniques required by wristed instruments demand a hybrid skill set. Studies have predictably demonstrated that experienced surgeons tend to obtain better results with traditional instruments in comparison to the same task using new surgical instruments (6). In order to adapt to diversifying practice, there is a need for a training period for the surgeon to attain the required competence.

Views currently remain dependent upon the quality of the camera, as well as the experience of the operating assistant. This is an aspect that is negated with a robotic system, as the views are entirely controlled by the lead surgeon. The procedure also benefits from the three-dimensional visualization at the console, enabling greater depth perception. In recognition of this, developers have strived to make a surgeon controlled “robotic” camera holder as well as a 3D VATS style camera.

## Conclusion

The next decade will see vast changes in the practice of thoracic surgery, with ongoing evolution of minimally invasive approaches. As surgeons we should embrace new technology to improve patient outcomes. Wristed instruments such as the ArtiSential® offer the benefits of robotic surgery, without the associated drawbacks. Our early experience has highlighted their exciting potential. By combining wristed instruments with the latest surgeon-controlled 3D camera technology, surgeon-powered robotics is an affordable reality.

## Data Availability Statement

All datasets generated for this study are included in the article/Supplementary Material.

## Author Contributions

JT and NC: wrote the original draft. All authors had a significant role in the conceptualization, data collection/literature review, and involved in reviewing and editing the final manuscript.

## Conflict of Interest

JD received consulting fees for ArtiSential® in the development of these instruments for thoracic surgery and was provided these instruments free of charge as part of an IRAS approved cohort study. The remaining authors declare that the research was conducted in the absence of any commercial or financial relationships that could be construed as a potential conflict of interest.

## Supplementary Material

The Supplementary Material for this article can be found online at the following links

Supplementary Video 1 Right lower lobectomy (https://youtu.be/K24NV8v22V4).

Supplementary Video 2 Left upper lobectomy (https://youtu.be/LiE_2mgQyHc).

Supplementary Video 3 Thymectomy (https://youtu.be/fPHXPzDI_0U).

Supplementary Video 4 Thymectomy with left SVC (https://youtu.be/ajk4HpmMR18).

Supplementary Video 5 Diaphragm plication (https://youtu.be/2d8pzqtSqvU).
